# Community Quarantine to Interrupt Ebola Virus Transmission — Mawah Village, Bong County, Liberia, August–October, 2014

**Published:** 2015-02-27

**Authors:** Tolbert Nyenswah, David J. Blackley, Tabeh Freeman, Kim A. Lindblade, Samson K. Arzoaquoi, Joshua A. Mott, Justin N. Williams, Cara N. Halldin, Francis Kollie, A. Scott Laney

**Affiliations:** 1 Liberia Ministry of Health and Social Welfare; 2 Epidemic Intelligence Service, CDC; 3 Bong Mines Medical Center, Bong Mines, Liberia; 4 Influenza Division, National Center for Immunization and Respiratory Diseases, CDC; 5 Division of Global Health Protection, Center for Global Health, Division of Global Health Protection, CDC; 6 Division of Respiratory Disease Studies, National Center for Occupational Safety and Health, CDC; 7 Fuamah District Health Team, Handii, Liberia

On September 30, 2014, the Bong County health officer notified the county Ebola task force of a growing outbreak of Ebola virus disease (Ebola) in Mawah, a village of approximately 800 residents. During September 9–16, household quarantine had been used by the community in response to a new Ebola infection. Because the infection led to a local outbreak that grew during September 17–20, county authorities suggested community quarantine be considered, and beginning on approximately September 20, the Fuamah District Ebola Task Force (Task Force) engaged Mawah leaders to provide education about Ebola and to secure cooperation for the proposed measures. On September 30, Bong County requested technical assistance to develop strategies to limit transmission in the village and to prevent spread to other areas. The county health team, with support from the Task Force and CDC, traveled to Mawah on October 1 and identified approximately two dozen residents reporting symptoms consistent with Ebola. Because of an ambulance shortage, 2 days were required, beginning October 1, to transport the patients to an Ebola treatment unit in Monrovia. Community quarantine measures, consisting of restrictions on entering or leaving Mawah, regulated river crossings, and market closures, were implemented on October 1. Local leaders raised concerns about availability of medical care and food. The local clinic was reopened on October 11, and food was distributed on October 12. The Task Force reported a total of 22 cases of Ebola in Mawah during September 9–October 2, of which 19 were fatal. During October 3–November 21, no new cases were reported in the village. Involving community members during planning and implementation helped support a safe and effective community quarantine in Mawah.

## Investigation and Results

In late August 2014, a male student (source patient) aged 22 years from Kakata, Liberia, the seat of Margibi County, traveled 20 miles (32 km) to Bong Mine Town in neighboring Bong County. He had signs and symptoms of Ebola, including fever, vomiting, and diarrhea, and was reported to have stayed overnight with family in Bong Mine Town on August 30. On August 31, he was taken by motorbike to his mother’s ancestral home in Mawah, 10 miles (16 km) by road. On September 1, he returned to Bong Mine Town, where he received home-based care for 3 days from a nurse-aid, before dying on September 4. No specimen was collected for Ebola virus testing, and he was buried in Bong Mine Town on September 6 by family members, who had not received training on safe burial practices.

The nurse-aid who provided care to the source patient in Bong Mine Town departed from there on September 3 and traveled to nearby Monokparga and Kalikata Meca, where he became ill. The nurse-aid continued to provide patient care after symptom onset, becoming the likely source of infection for a cluster of at least nine Ebola cases. Currently, no additional information is available regarding this separate cluster.

On September 9, an adult male Mawah resident who reportedly had contact with the source patient during his overnight stay in Mawah developed Ebola-like symptoms. He tested positive for Ebola virus, and later died. During September 9–16, six households of his and the source patient’s contacts were quarantined in an effort to prevent transmission within the village, and to prevent spread to other areas. Six additional persons experienced onset of symptoms during September 17–20, and each later died.

**FIGURE 1:**
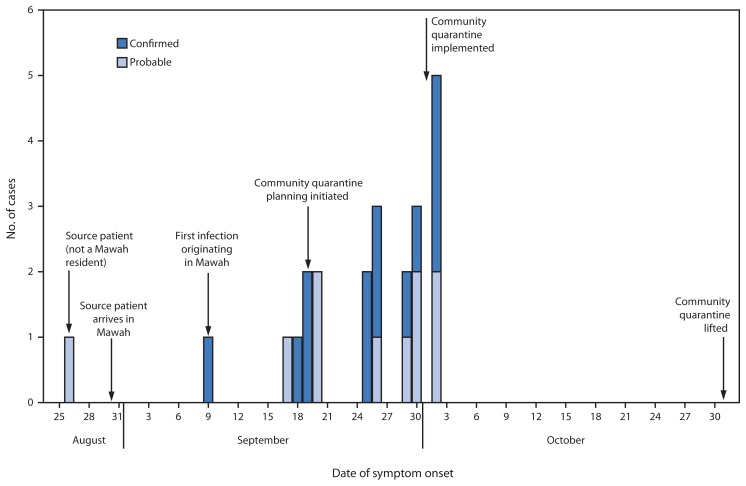
Number of probable and confirmed Ebola virus diseases cases, by date of symptom onset — village of Mawah in Bong County, Liberia, August 26–October 31, 2014

An investigation by the Task Force identified 22 incident cases of Ebola (13 confirmed and nine probable) in Mawah (Figure 1), resulting in 19 deaths during September 9–October 2. Seven of the patients were female, and the median age was 44 years. A total of 160 contacts were identified.

## Public Health Response

Because of the increase in cases, on approximately September 20, the county health officer suggested that the Task Force chair (TFC), a local physician, consider community quarantine as an additional measure. After multiple meetings with the village chief and elders to provide information on Ebola and its transmission, and inclusion of the paramount chief (ranking traditional leader) in the Task Force, the TFC proposed a community quarantine period of 21 days, consisting of the following measures:

1. Restrictions on residents leaving Mawah (e.g., checkpoint at access road),
2. Prohibition of nonresidents entering Mawah (e.g., road checkpoint),
3. Regulation of local river crossings, and
4. Closure of two local markets.

Local leaders expressed two main concerns relating to the proposal. First, the measures would leave the village without important food and income sources because residents fish in the St. Paul River and rice fields are on the opposite bank. Leaders agreed it was prudent to regulate cross-river traffic, but were concerned that residents’ livelihoods would suffer if fishing grounds and fields were off-limits, and if markets were closed. Second, community leaders were concerned that the proposed measures would limit residents’ access to basic medical services. A clinic normally operated in Mawah, but it had closed recently, leaving a clinic in a neighboring village as the only remaining option within walking distance.

**FIGURE 2:**
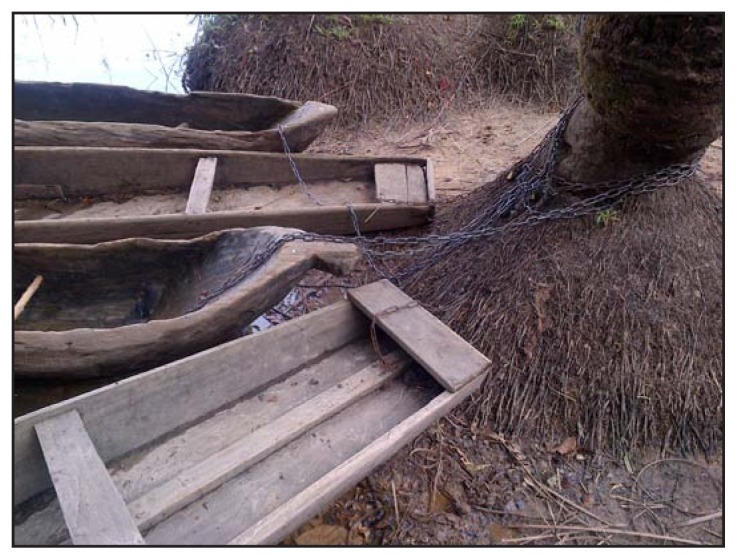
Most community canoes were chained to trees for the duration of the community quarantine period — village of Mawah in Bong County, Liberia, 2014 Photo/David J. Blackley

Community quarantine was instituted on October 1 and presented the community and the Task Force with challenges. For example, the reopening of the clinic and food delivery were delayed by approximately 1 week while county and international partners requested and coordinated the needed personnel and resources. During the first week of October, the Task Force requested support to address issues relating to medical care and food. To reopen the medical clinic, Bong County paid a nurse to manage the clinic 2 days per week, and an international nongovernmental organization provided three support staff, which allowed services to resume on October 11. The nurse was provided a noncontact infrared thermometer and was instructed to avoid providing care to persons with symptoms consistent with the case definitions for suspected or probable Ebola, but rather to seek support in arranging for safe ambulance transport. Partners in Bong County contacted the World Food Programme, which on October 12 delivered a 45-day ration of rice, corn, beans, and lentils, and assisted village leaders with distribution. During the quarantine, members of the Task Force visited the village at least twice per week to provide psychosocial support for affected families, with special emphasis on survivors and those with family members receiving treatment in an Ebola treatment unit. The TFC worked with local leaders to arrange regulated cross-river transport so residents could access fields for harvest. The crossing was open to farmers each morning, closed during the middle of the day, and then reopened each evening to allow return. Hand hygiene supplies were placed at the crossing, and two canoe pilots were identified to provide the only river crossing services. Canoes not belonging to approved pilots were chained to trees (Figure 2). Pilots were instructed to deny transport to symptomatic persons and to asymptomatic nonresidents (e.g., persons attempting to reach a market). After the safe transport of symptomatic persons to an Ebola treatment unit during October 1–2, and institution of community quarantine, Mawah had no newly reported cases for the remainder of the quarantine period, which was extended by 10 days as a precautionary measure and concluded on October 31.

### Discussion

Community quarantine is controversial, and implementation requires careful consideration of the balance between public health and individual rights (1). Potential secondary consequences, including insufficient access to food and medical care, are important considerations. Although no causal conclusions can be drawn from the Mawah experience about the effectiveness of community quarantine, it illustrated a number of issues that must be addressed in such situations.

First, community leaders needed to be convinced that the disease was real. During the current Ebola epidemic, earning community trust and confidence in response efforts has at times been challenging (2). In Mawah, community members might only have been willing to accept the proposed quarantine after witnessing the devastating effect Ebola had on the village. Second, a trusted local leader with health expertise and an understanding of the culture acted as a liaison between community leaders and district health authorities. Communities might benefit from formal integration of traditional leaders into outbreak response planning, and could consider offering both traditional and political leaders opportunities to provide feedback before decisions are made relating to proposed public health interventions. Third, local leaders worked to ensure that basic needs (e.g., food, medical, and psychosocial) were met for the duration of the quarantine period. In this situation, support was provided by the county and international nongovernmental organizations. In other low-resource settings, or in areas with small populations, a similar approach might be necessary to ensure that community needs are met if community quarantine is determined to be an effective approach to interrupting Ebola virus transmission. Finally, the appropriate isolation of sick persons and comprehensive contact tracing remain essential components of an Ebola response, irrespective of decisions on community quarantine.

What is already known on this topic?

Community quarantine can be controversial and logistically difficult to implement.

What is added by this report?

During September–October 2014, multiple partners responded to an outbreak of Ebola virus disease (Ebola) in the village of Mawah in Bong County, Liberia; county officials proposed community quarantine. Local traditional leaders were integrated into response planning and raised concerns about availability of medical care and food. Community quarantine was implemented, and local, national, and international partners arranged to reopen a local clinic, deliver food, and provide psychosocial support. After removal of symptomatic patients and implementation of community quarantine, Mawah reported no new Ebola cases.

What are the implications for public health practice?

Community quarantine in a low-resource setting can restrict access to critical goods and services. Involving local leaders during planning and implementation can help ensure community needs are met. Isolation of ill persons and contact tracing remain essential components of an Ebola response, irrespective of decisions on community quarantine.
